# Supplementary material for: The genome sequence of the Purple-shot Copper, Lycaena alciphron (von Rottemburg, 1775) (Lepidoptera: Lycaenidae)
Source: Wellcome Open Res. 2025 Jul 28;10:375. [Version 1] doi: 10.12688/wellcomeopenres.24617.1 (PMC12572776; doi:10.12688/wellcomeopenres.24617.1)
Supplement: Peer review history

# Reviewer report: Reviewer response for version 1

**DOI:** 10.21956/wellcomeopenres.27127.r134611

**Published:** 2025-10-29

**Authors:** Sara E Mousa, Gehad N Aboulnasr

**Affiliations:** 1 Plant Protection Department, Faculty of Agriculture, Assiut University, Assiut, Egypt; 2 Zoology and Entomology Department, Faculty of Science, Assiut University, Assiut, Egypt

The most important aspects of this paper, which is a **Data Note** presenting a new genomic resource, are:

 1. The Creation of a High-Quality Genomic Resource

 The central importance lies in the successful generation of the **first genome assembly** for the **Purple-shot Copper butterfly, *Lycaena alciphron***.

- **Chromosome-Level Assembly:** The research produced a **chromosome-level, haplotype-resolved reference genome**. This means the assembled DNA sequences are not just fragments but are organized into the correct 25 chromosomes (23 autosomes, Z, and W sex chromosomes).
- **High Quality Standard:** The assembly meets the demanding **Earth BioGenome Project (EBP) reference standard** (6.C.Q62), with an exceptionally high quality value ( **QV of 62.2**), ensuring its reliability for future studies.

2. Significance for Evolutionary Biology

 The primary utility of this dataset is in advancing the study of insect evolution.

- **Chromosomal Evolution:** The genome provides a crucial foundation for investigating the **patterns of chromosomal evolution** and changes in linkage groups (Merian elements) within the Lepidoptera (butterfly and moth) order.
- **Species-Specific Resource:** As the first of its kind for *L. alciphron*, it provides tools for population genetics, conservation biology, and identifying genes related to key traits in this specific species.

3. Technical Soundness and Accessibility

 The importance is reinforced by the methods used and the accessibility of the final data.

- **Technical Rigor:** The work is deemed **technically sound** with appropriate protocols, ensuring the reliability of the assembly.
- **Replicability:** Sufficient details on methods and materials are provided, making the work **fully replicable**.
- **Accessibility:** The final dataset is publicly available through the **European Nucleotide Archive (ENA)**, ensuring the genome is a lasting and accessible resource for the global scientific community.

Are sufficient details of methods and materials provided to allow replication by others?

Yes

Is the rationale for creating the dataset(s) clearly described?

Yes

Are the datasets clearly presented in a useable and accessible format?

Yes

Are the protocols appropriate and is the work technically sound?

Yes

Reviewer Expertise:

Entomology, Molecular Genetics, Ecology, Biological Control, Plant Protection

We confirm that we have read this submission and believe that we have an appropriate level of expertise to confirm that it is of an acceptable scientific standard.

# Reviewer report: Reviewer response for version 1

**DOI:** 10.21956/wellcomeopenres.27127.r134616

**Published:** 2025-10-06

**Authors:** Jason Charamis

**Affiliations:** 1 Foundation for Research and Technology - Hellas, Irákleion, Greece

This manuscript reports the first genome assembly of *Lycaena alciphron. *The work is technically sound and the methods used are clearly described. My only suggestion is to also add a gene annotation to this assembly, which -as described- in the Data Availability section will be performed through Ensembl at the European Bioinformatics Institute.

Are sufficient details of methods and materials provided to allow replication by others?

Yes

Is the rationale for creating the dataset(s) clearly described?

Yes

Are the datasets clearly presented in a useable and accessible format?

Yes

Are the protocols appropriate and is the work technically sound?

Yes

Reviewer Expertise:

Arthropod Comparative Genomics

I confirm that I have read this submission and believe that I have an appropriate level of expertise to confirm that it is of an acceptable scientific standard.
